# Predicting repeated self-harm or suicide in adolescents and young adults using risk assessment scales/tools: a systematic review protocol

**DOI:** 10.1186/s13643-019-1007-7

**Published:** 2019-04-04

**Authors:** Isobel Marion Harris, Sophie Beese, David Moore

**Affiliations:** Institute of Applied Health Research, Public Health Building, University of Birmingham, Birmingham, B15 2TT UK

**Keywords:** Self-harm, Suicide, Predictive ability, Adolescents

## Abstract

**Background:**

Self-harm and suicide have been identified as serious public health problems in children, adolescents, and young people across the world. Suicide is a major cause of mortality in this population and is commonly preceded by self-harm. Both suicide and self-harm are difficult to predict, and several risk scales and tools are in use for this purpose. Currently, there is only a small amount of evidence available regarding their predictive ability in clinical practice, and no consensus as to which is the most suitable for particular populations or settings. The aim of this review is to evaluate the ability of risk scales to predict future episodes of suicide or self-harm in adolescents and young adults presenting to clinical services with attempted suicide or an episode of self-harm.

**Methods:**

A comprehensive search of electronic databases (MEDLINE, EMBASE, CINAHL, and PsycINFO) from inception will be conducted to identify studies that look at the ability of risk scales to predict suicide or future episodes of self-harm in adolescents and young adults presenting to clinical services with attempted suicide or an episode of self-harm. Two authors will independently carry out key methodological steps such as study screening and selection and data extraction. Quality assessment will be carried out using a checklist developed from the QUIPS and QUADAS-2 tools. Data will be grouped by tool and a narrative synthesis undertaken. For each tool, meta-analysis will be undertaken for ability to predict suicide or repeat self-harm where clinical and methodological homogeneity exists.

**Discussion:**

This systematic review will be the first to explore the use of assessment scales/tools in an adolescent population and will help to inform current practice regarding scales/tools with higher predictive ability. There is currently no evidence specifically for this population and a clear need with a high prevalence of self-harm and suicide in adolescents. Additionally, this review will help guide future research into suicide and self-harm prediction and prevention.

**Systematic review registration:**

PROSPERO CRD42017058686

## Introduction

### Background

Self-harm and suicide have been identified as serious public health problems in children, adolescents, and young people aged 25 years and under across the world [1–3]. The term self-harm can be used to refer to acts of self-poisoning or self-injury (e.g. cutting, scratching, breaking bones, burning) carried out intentionally regardless of motive or suicidal intent [1, 4, 5]. The prevalence of self-harm can be difficult to determine as it is usually a secretive and hidden behaviour [5]. Less than 20% of adolescents who self-harm seek treatment resulting in substantial unreported cases [6, 7]. Despite this, community-based studies have reported prevalences of 10–18% in adolescent populations [6–13], and these rates appear to be rising [14, 15]. Data from the UK shows that the highest rates of self-harm occur in adolescent and young adult populations and that there is marked gender disparity between those aged 16–24 [9, 11], with 25.7% of young women having self-harmed compared with 9.7% of young men [15]. Repetition of self-harm is common, with 15–25% of adolescents treated at hospital for an episode of self-harm return for treatment within 12 months [2, 16]. Repetition has also been shown to be common in those not engaging with clinical services [7].

There are 164,000 suicides per year worldwide in those aged less than 25 years, but this is thought to be vastly less than the true figure [17]. Whilst the prevalence of suicide in young people in the UK is low compared to the older population, it still remains a major mortality cause in young people [18, 19]. Adolescent suicide is suspected to be under-reported due to perceived stigma and misclassification [20]. Worldwide, it is the most common cause of death for female adolescents and the third most common for male adolescents after road traffic accidents and violence [17]. There is a strong association between self-harm and risk of future suicide [21, 22], with approximately 50% of adolescents who die by suicide having previously self-harmed [19]. Suicide prevention has been identified as a public health priority, with both national and global initiatives in place targeting early identification and risk management as key strategies to prevent suicide attempts and save lives [23, 24].

### Rationale

Both self-harm and suicide are difficult to predict in adolescents [1, 25]. The UK guidelines for the management of self-harm in those aged over 8 years recommend that all patients who present with self-harm should undergo comprehensive psychosocial assessment including assessing the risk of repetition of self-harm or suicide [4, 26, 27]. This recommendation was informed by evidence from a systematic review carried out as part of the guideline development process [4]. Risk scales/tools tend to be a key part of this assessment; however, there is currently only a small amount of evidence available regarding their use and effectiveness, and no guidance as to which to use or which is best for particular populations or settings [4, 28].

There is a large variation in the type, format, and content therein of risk scales/tools being used across the UK [29–31]. Some only assess a few parameters and others assess a more extensive range. The content of these tools includes previous self-harm, method of harm, current psychiatric treatment, age, and employment status [32–35]. Some are short, for example the Manchester Self-Harm Rule and the ReACT Rule assessing four and five parameters respectively. In contrast, the Repeated Episodes of Self-Harm (RESH) Score and the SADPERSONS Scale are longer, assessing about nine and 11 different parameters respectively [32–35].

There is a desire for consistent good practice with calls for further research and a recommendation from the Royal College of Psychiatrists UK that locally developed scales/tools should no longer be used [36]. A survey of 32 English hospitals found that over 20 different risk scales/tools were in use, many of these were locally developed, highlighting a lack of consistency of practice [28].

Scoping searches of Medline and the Cochrane library, without restriction on population age, identified two published systematic reviews [3, 37]. Both focus on an adult population and neither was able to conclude that any one scale performed better than another, or say with any certainty which scale had the best ability to predict future self-harm or suicide attempts. Both reviews agree that further work is required to explore the use of risk assessment scales/tools further as currently there is a paucity of robust evidence on the topic.

As neither review focused specifically on adolescents, and as this is a key age group affected by self-harm and suicide, a review targeting scales/tools for adolescents is clearly required. The scoping searches identified a body of published literature specific for this population supporting feasibility [38–42].

### Aim

This systematic review aims to evaluate the ability of risk scales to predict suicide or future episodes of self-harm in adolescents and young adults presenting to clinical services with attempted suicide or an episode of self-harm.

## Methods

This protocol has been prepared according to the Preferred Reporting Items of Systematic Reviews and Meta-Analysis Protocols (PRISMA-P) statement [43] and registered on PROSPERO (CRD42017058686).

### Eligibility criteria

The criteria outlined below will be used to select studies for inclusion in the review.

#### Study designs

Prospective and retrospective cohort studies, case-control studies, and randomised controlled trials testing assessment scales/tools.

#### Participants

The participants are adolescents who have self-harmed or attempted suicide.

Adolescence is a term that lacks a formal definition. It can refer to age, physical characteristics, or cognitive development [1]. The World Health Organisation defined adolescence as being between 10 and 19 years [44]; however, in the literature, the upper limit for adolescents can range from 18 to 25 years [1]. In an attempt to include all relevant data in this review, reflecting this and that self-harm is rare before the age of 12 [45], a broad range of adolescents and young people aged 10–25 who have self-harmed or attempted suicide will be included. Studies also containing older populations will be included if data for 10–25-year-olds is presented separately. If not presented separately, study authors will be contacted to try to obtain data for the subgroup before study exclusion. Studies containing adolescents who have and have not self-harmed or attempted suicide will be included. Where data on self-harm/suicide attempters is presented as a subgroup, only this data will be used; where the data is not presented as a subgroup, the study will only be included if 50% or greater of the population are self-harm/suicide attempters. This is an arbitrary threshold. The effect of such studies on the analysis will be explored in sensitivity analyses if appropriate.

#### Intervention/assessment

Any risk assessment carried out on patients who have presented to clinical services or been treated by a healthcare professional. No restrictions will be placed regarding the content of the assessments.

#### Setting

Risk assessment must be carried out within a clinical setting or by a health care professional. This can be an inpatient or outpatient facility or as part of a home treatment program.

#### Timing

There will be no restriction on the length of follow-up so long as outcomes occur after the assessment is carried out.

#### Outcome

In repeat self-harm and/or attempted suicide or completed suicide, it is acknowledged that these may be recorded individually or grouped together as a composite outcome. Strategies for dealing with this are outlined further in the protocol.

#### Language

No language restrictions will be put in place.

### Search strategy

Electronic searches of bibliographic databases (MEDLINE, EMBASE, CINAHL, PsycINFO, and Open Grey) will be carried out with a search strategy developed using where appropriate index and free-text terms related to self-harm, suicide, adolescents, and risk assessment. Terms for self-harm, suicide, risk, prediction, and adolescents will be combined using the AND operator. The MEDLINE search strategy is presented in Table 1. The strategy will be adapted to the syntax and subject headings of each database. Articles cited by included studies will be checked to identify any additional studies.

**Table 1:** Example search strategy for MEDLINE

1	((self or themsel$ or onesel$) adj2 (harm$ or cut$ or immolat$ or inflict$ or injur$ or mutilat$ or poison$ or damag$ or destruct$)).ti,ab.
2	overdos$.ti,ab.
3	Self-Injurious Behavior/
4	Self-Injurious Behavio?r.ti,ab.
5	Self Mutilation/
6	Suicide/
7	Suicide, Attempted/
8	Suicidal Ideation/
9	(suicid$ not (assisted adj suicide$)).ti,ab.
10	((auto adj (aggress$ or mutilat$)) or (autoaggress$ or automutilat$)).ti,ab.
11	(parasuicid$ or para-suicid$).ti,ab.
12	1 or 2 or 3 or 4 or 5 or 6 or 7 or 8 or 9 or 10 or 11
13	Suicide, Assisted/
14	12 not 13
15	Risk Factors/
16	Risk/
17	(risk or predict$ or prognos$ or assess$).ti,ab.
18	15 or 16 or 17
19	exp psychiatric status rating scales/
20	severity of illness index/
21	exp personality assessment/
22	(inventor$ or checklist$ or scale$ or rating or model$ or tool$ or rule or questionnaire$ or index$ or indices).ti,ab.
23	19 or 20 or 21 or 22
24	18 and 23
25	“adolescen$”.ti,ab.
26	Adolescent/
27	Young Adult/
28	Child/
29	25 or 26 or 27 or 28
30	14 and 24 and 29

### Study records

#### Data management

Results from the literature search will be imported into EndNote X7 software (Clarivate Analytics), allowing automatic and manual identification and removal of duplicate records.

#### Selection process

The titles and abstracts will be screened for relevance against the selection criteria. Full text of relevant articles will be obtained and selected for review against the full inclusion criteria. Clarification will be sought from study authors where possible if eligibility cannot be determined. Two reviewers will independently undertake the screening process, and disagreements will be resolved by discussion and, if required, involving a third person. Reasons for articles not meeting the inclusion criteria will be recorded. Translations will be sought for languages other than English to aid selection and/or subsequent reviewing. If no translation can be obtained, articles will be reported as non-assessable rather than as excluded. The article selection process will be fully reported in the PRISMA flow diagram format [46].

#### Data collection process

Two reviewers will independently extract data from each article using a piloted extraction form. A third independent person will be consulted to resolve disagreements. Data will be managed using Excel 2010 (Microsoft). Data to be extracted will include the following: study characteristics (duration, start and end date, country and setting), participant characteristics (number, average age, gender, ethnicity, socio-economic details, co-morbidities), any reported subgroups, scale/tool used, content of scale/tool, if scale/tool is validated, scale/tool development details, data collection method, outcome(s) measured (e.g. repeat self-harm), method of outcome assessment, total number followed up, loss to follow-up number and reasons, number of events for each outcome and data on measures of association between assessment tool and outcome (e.g. relative risk, sensitivity, specificity) along with attendant precision (95%CI, *p* values), or raw data to calculate these. Unadjusted and adjusted data along with factors adjusted for will be recorded. If any data are found to be missing, the corresponding author of the article will be contacted by email. One email and one follow-up email will be sent if no response is received to the first email.

#### Quality and risk of bias assessment

Quality and risk of bias assessment of articles will be carried out by two reviewers independently. A checklist modified from Quality in Prognostic Studies (QUIPS) [47, 48] and Quality Assessment of Diagnostic Accuracy Studies-2 (QUADAS-2) [49], checklists suitable for prognostic and diagnostic tests, will be used. This is appropriate for this review as the inclusion of studies that may have prognostic or diagnostic features, and the nature of the data to be extracted, means that neither checklist individually would completely assess all important elements. These tools assess bias risk in several domains including the selection of patients and reporting of outcomes.

## Analysis

A narrative synthesis will be carried out presenting information from all included studies to explore similarities, differences, and findings within and between them. Information will be presented in a variety of formats including tables, figures, and text. The content of tools/scales will be reported and compared. Where possible, studies will be grouped and analysed by scale/tool for both descriptive and numeric data. Each scale/tool will be considered separately with regards to the outcomes of repeat self-harm, attempted suicide, or completed suicide. Potentially, there may be discrepancies between studies in how outcomes are recorded and that studies may not record the three outcomes separately as considered by this review. For example, one study may record self-harm as attempted suicide and another study may record the same behaviour as self-harm, or a study may just look at one outcome comprising all behaviours.

Additionally, completed suicide is a rare outcome, and therefore, it may be that there are not many reports of this outcome or a zero event rate will be reported. Completed suicide, attempted suicide, and/or a composite outcome will be considered separately.

Depending on the data available, it may be possible to consider the prognostic ability (how well the scale/tool can predict each individual patient that will repeat harm/suicide) of each scale/tool. A decision will be made about whether to consider prognostic ability will be made after data extraction has been carried out.

It is possible that prognostic models have been developed either using data collected from a checklist or the checklist derived from a model. Therefore, it would be appropriate to assess these using tools appropriate for models. Assessment of models involves consideration of the development of the model and the consistency of performance and decision-making of the model [50]. Studies reporting prognostic models may be reporting either model development, validation of the model, or both [51]. If studies reporting models are included, it may be appropriate to extract further data regarding the stage of model development/validation.

### Data synthesis

Data synthesis and analysis will be carried out using Excel 2010 (Microsoft).

For each tool, outcome (e.g. self-harm), and outcome measure (e.g. relative risk) grouping, contributing studies, and data will be examined for clinical and methodological homogeneity. This will inform a decision on whether a meta-analysis is appropriate and, if so, whether a fixed or random effects model is used. However, it is expected that the random effects model will be most appropriate. The degree of statistical heterogeneity will be assessed using the *I*^2^ statistic. Where meta-analysis is not deemed appropriate, data may be presented in forest pots without a summary estimate. This will include data for each outcome using the same outcome measure across all tools, to illustratively show all such data on a single plot.

Subgroup analyses will be carried out if feasible and warranted to examine potential effect modifiers based on gender, setting (e.g. inpatient vs outpatient), and patients presenting after a first occurrence of self-harm/attempted suicide versus patients presenting after a repeated occurrence, or patients who are assessment-naïve versus patients who have had risk assessments carried out before. Further analysis will also be carried out if possible grouping studies by tool intent, i.e. those that are specific for self-harm/suicide and those that are non-specific (e.g. general depression screening tools). These are, however, very dependent on the data being present in the included studies, and it is recognised that this may not be possible.

Sensitivity analyses will be carried out to assess the impact of decisions taken during the review if feasible to explore the effects of age (over and under 18 years) and risk of bias. The sensitivity analyses around age are of particular relevance and interest to this review due to the previously mentioned lack of formal definition for the term adolescence. Analyses in this area would offer more insight across the age range as there is a wide difference between the lower and upper limits of the 10–25-year range included in this review in terms of life stage and development, as well as purely chronological age.

If more than ten studies are included in a single meta-analysis, a funnel plot will be used to explore the potential for biases including publication bias.

The methodological quality of the contributing studies will be considered in the context of each outcome.

## Discussion

This will be the first review to investigate the use of risk scales/tools in an adolescent population and will contribute to the body of evidence for self-harm and suicide prediction and prevention. Understanding of how to predict self-harm and suicide in the population most at risk is crucial to tackling this major public health problem and provide an evidence base to ensure that informed decisions can be made regarding predication of future risk.

This review is needed as there is currently no systematic review examining risk scales/tools in an adolescent population, and this has been identified as a key age group affected by self-harm and suicide. Specifically focusing on this age group will allow greater understanding of the use of risk assessments in this population. Additionally, the documented wide variety in the type of risk scales/tools being used across hospitals and current lack of guidance surrounding their use in particular patient groups and settings (including hospitals, outpatient, and home treatment settings) warrants further investigation to improve and standardise patient care. The lack of consensus mentioned previously shows that more knowledge is required in this area to address the needs of high-risk patients and to try to identify a gold standard for assessment of these patients.

The findings will be of benefit to a variety of stakeholders as it will provide knowledge to guide best practice, providing clarity to healthcare professionals and benefit to patients, and highlight areas requiring further research in this population.

## Abbreviations

PRISMA: Preferred Reporting Items for Systematic Reviews and Meta-Analyses
PRISMA-P: Preferred Reporting Items for Systematic Review and Meta-Analysis-Protocols
QUADAS-2: Quality Assessment of Diagnostic Accuracy Studies-2
QUIPS: Quality in Prognostic Studies
